# Experiences as a clinical teaching fellow: interviews with clinical teaching fellows in the West Midlands

**DOI:** 10.1186/s12909-024-05958-2

**Published:** 2024-09-16

**Authors:** Isobel Marion Harris, Nicola Dennis, Derek J Ward, Alice J Sitch, Jayne Parry, Sheila Greenfield

**Affiliations:** 1 Institute of Applied Health Research, University of Birmingham, Birmingham, UK; 2 National Institute for Health and Care Research (NIHR) Birmingham Biomedical Research Centre, Birmingham, UK

**Keywords:** Teaching fellows, Undergraduate medical education, Workforce, Teaching, Interviews

## Abstract

**Background:**

The majority of junior doctors in the UK do not proceed directly into specialty training after completing mandatory foundation training but instead take a year out of training. A common post undertaken during a year out of training is a clinical teaching fellow (CTF) role which is used to provide undergraduate medical student teaching. There is only a small amount of literature available regarding CTF posts, and very little of this explores experiences or reasons for taking up such as post. An understanding of the reasons why doctors are choosing to work as CTFs and what their experiences are in post will contribute to how the role is further developed and utilised within the NHS. This study aimed to explore the experiences of CTFs employed in the West Midlands at NHS hospital Trusts.

**Methods:**

CTFs working in Trusts in the West Midlands region registered as students on the Education for Healthcare Professionals Post Graduate Certificate course at the University of Birmingham in August 2019 and 2020 who were enrolled in a longitudinal study were invited to take part in an individual interview asking about their experiences as CTFs.

**Results:**

Nine CTFs participated in an interview. Five main themes were identified which related to their experiences in post and plans for future careers. Participants reported choosing to undertake a CTF role due to wanting a break from clinical work and having previously enjoyed delivering teaching. Positive experiences in post included lifestyle related benefits and self-development opportunities. Challenges identified with the role included the impact of COVID-19 and volume of students.

**Conclusion:**

This is the first study to use interview methodology to explore experiences of CTFs, and has provided a valuable insight into the experiences of those in post in the West Midlands region. Understanding why doctors chose this job and what their experiences are could help to further develop and refine the role. To guarantee demands for teaching staff are met those employing CTFs should be aware of reasons why doctors apply for the post and ensure the post remains a desirable option.

**Supplementary Information:**

The online version contains supplementary material available at 10.1186/s12909-024-05958-2.

## Introduction

In the UK, the majority of junior doctors do not proceed directly into speciality training programmes after completing the mandatory two years of Foundation training [1]. Taking a year out of training, which can be referred to as an “F3 year” [2] or a ‘post-foundation training break’ (PFTB) [3], is now commonplace and there are a variety of reasons for choosing to do this including wanting a break from providing clinical service in a training programme, uncertainty regarding future specialty, wellbeing factors, and wanting to pursue other opportunities [2]. For the majority of doctors, taking an F3 year/PFTB is a choice rather than a requirement as a result of not gaining a specialty training place [3].

One role commonly undertaken during an F3 year is working in a clinical teaching fellow (CTF) post. CTF roles tend to be one year in length and primarily involve delivering hospital-based teaching to undergraduate medical students [4], although a small number of posts are available delivering teaching in universities [5]. Individual CTF posts vary across the country, and even within the same hospital, with roles having differences in time spent delivering teaching and clinical service provision, as well as other duties that can include undertaking research, and providing pastoral support to students [4, 6–8]. Most CTFs roles also offer the opportunity to obtain postgraduate qualifications related to education [4].

There is no record of how many CTFs posts are available across the UK as they are employed locally by individual NHS Trusts or medical schools and there is no standardisation of the role. It is known however that the number of CTF posts has increased over recent years. In 2005, 77 CTF posts were recorded across the UK [8], however a study in 2018 reported 101 posts in one geographical area of the UK alone (North East England), with 26 of those employed at a single hospital Trust [7].

The number of medical student places available in the UK has expanded over recent years in response to concerns about NHS understaffing and the future increased demand on hospital services. There are currently approximately 9,500 medical places available across the UK [9], and it has been announced that the total number of medical school places will be increased to 15,000 per year by 2031/32 as part of the NHS Long Term Workforce Plan [10]. An increased number of medical students will require an increased educational workforce in order to deliver adequate levels of teaching, and it can be speculated that this could be a reason for the increase in the number of CTF posts available although here is no formal documentation of this.

There is only a small amount of literature available regarding the CTF role which is primarily career advice/opinion pieces [4, 11–17] and are usually written by those who have previously held a CTF role. A small number of additional primary studies exist which have attempted to map out the CTF role [8], evaluate the CTF role from the point of view of undergraduate medical students [18], document challenges of the post from the perspective of CTFs [7], and we have previously explored the views of senior hospital doctors in the West Midlands regarding CTFs [19] and the expectations of CTFs in the West Midlands [20]. There is currently no literature exploring why doctors choose to delay specialty training to work in a CTF role, what their experiences of the role are beyond challenges faced [7], or whether and how their expectations of the year in post were met. No previous study has used interview methodology to explore the experiences of CTFs.

As the role continues to grow in popularity, a deeper understanding of the reasons why doctors are choosing to work as CTFs and how the post meets their expectations for the year is warranted. This will help to ensure that posts are meeting the needs of both the CTFs and the Trusts that employ them meaning that maximum benefit can be derived from the role. This paper presents the results of in-depth interviews with CTFs who were recruited as part of a longitudinal study run over two years to reflect on their experiences in post. The expectations and experiences of participants from the first year of the study have been reported separately [20], and this paper reports the data from participants from both years of the study who were invited to take part in an interview.

## Methods

### Objective

To explore the experiences of CTFs in one region of England at the end of their year in post.

### Participants

CTFs working in Trusts in the West Midlands region registered as students on the Education for Healthcare Professionals Post Graduate Certificate (PGCert) course at the University of Birmingham (UoB) in August 2019 and 2020 who were enrolled in a longitudinal study were invited to take part in an individual interview. Two reminder emails were sent to participants who did not respond. Participants who did respond were emailed the Participant Information Sheet and a consent form to fill in ahead of the interview.

### Data collection

Interviews took place between June and July 2021, and were all held virtually via Zoom [21]. The interviews lasted between 21 and 50 min and were recorded using Zoom’s recording and automatic transcription functionalities. Each interview was conducted using the same topic guide (appendix 1) and explored the participants’ experiences of their time as a CTF by asking broadly about their year in post, why they had wanted to do the role, and how they felt having done the role would impact upon their future career. All participants were interviewed by IH, a doctoral researcher and research fellow in Applied Health Research at the University of Birmingham (UoB).

### Data analysis

The interview transcripts were analysed using thematic analysis [22] which involved coding the transcripts then identifying themes in the data. An inductive approach was taken as the interviews were exploratory in nature [23]. All transcripts were independently coded by two researchers (IH and ND, a Public Health Registrar with an interest in medical education) and meetings were held to discuss coding, check agreement, and to resolve any differences through discussion. Additional regular meetings were held with other researchers (JP, SG, DW, AS) to discuss and refine coding and analysis.

### Ethical considerations

Ethical approval was granted by UoB in January 2020 (ERN_19–0687). Subsequent amendments were made by chair decision (ERN_19-0687 A) or approved in line with the UoB Research Ethics and Governance Exceptional Circumstances Due to COVID 19 guidance.

## Results

Of the 31 participants who had taken part in the longitudinal study, nine (29%) indicated they were willing to take part in an interview (two recruited from the first year, and seven from the second year of the original longitudinal study).

Participant characteristics of the nine participants are shown in Table 1. The nine participants (two male and seven female) were based at six different Trusts, five worked full time, and four part time. All the participants had studied an undergraduate MBChB course and five participants had undertaken an intercalated degree during their studies. Four participants were contracted to do clinical work as part of their job and the other five were not. Of these five, three had the option to work half a day per week in a supernumerary clinical role of their choice if they wanted, but this was not mandatory.

**Table 1:** Participant characteristics

Characteristic	Participant *n* (%)
Male/female	
*Male*	2 (22)
*Female*	7 (78)
MBChB course studied	
*Undergraduate*	9 (100)
*Graduate*	0 (0)
Years after qualifying	
*Two years*	4 (44)
*Three-four years*	1 (11)
*Five + years*	4 (44)
Prior degree	1 (11)
Intercalated degree	5 (56)
*Biosciences subject*	*3 (60)*
*Humanities subject*	*2 (40)*
Previous teaching qualification	1 (11)
Full time CTF post held	5 (56)
Contracted clinical work part of role	
*Yes*	4 (44)
*No*	5* (56)

*3 participants had the option to work half a day per week in a supernumerary clinical role if they wished but it was not contracted

In order to maintain the confidentiality of participants, identifiable details such as the name of Trust worked at and/or any job specific information have been removed from quotes provided in this section. Table 2 provides individual characteristics of each participant to provide background information.

**Table 2:** Individual participant characteristics

Participant number	First/second year of study	Male/female	Years after qualifying	Full/part time post	Trust worked at
1	First	Male	Five+	Part time	a
2	First	Female	Five+	Part time	b
3	Second	Male	Two	Full time	c
4	Second	Female	Five+	Full time	d
5	Second	Female	Two	Full time	e
6	Second	Female	Two	Part time	b
7	Second	Female	Two	Full time	b
8	Second	Female	Five+	Full time	a
9	Second	Female	Three-four	Part time	f

Five main themes were identified from the data. These were:

1. Why are we doing this?
2. The role.
3. Advantages.
4. Challenges.
5. What’s next?

**Fig. 1:**
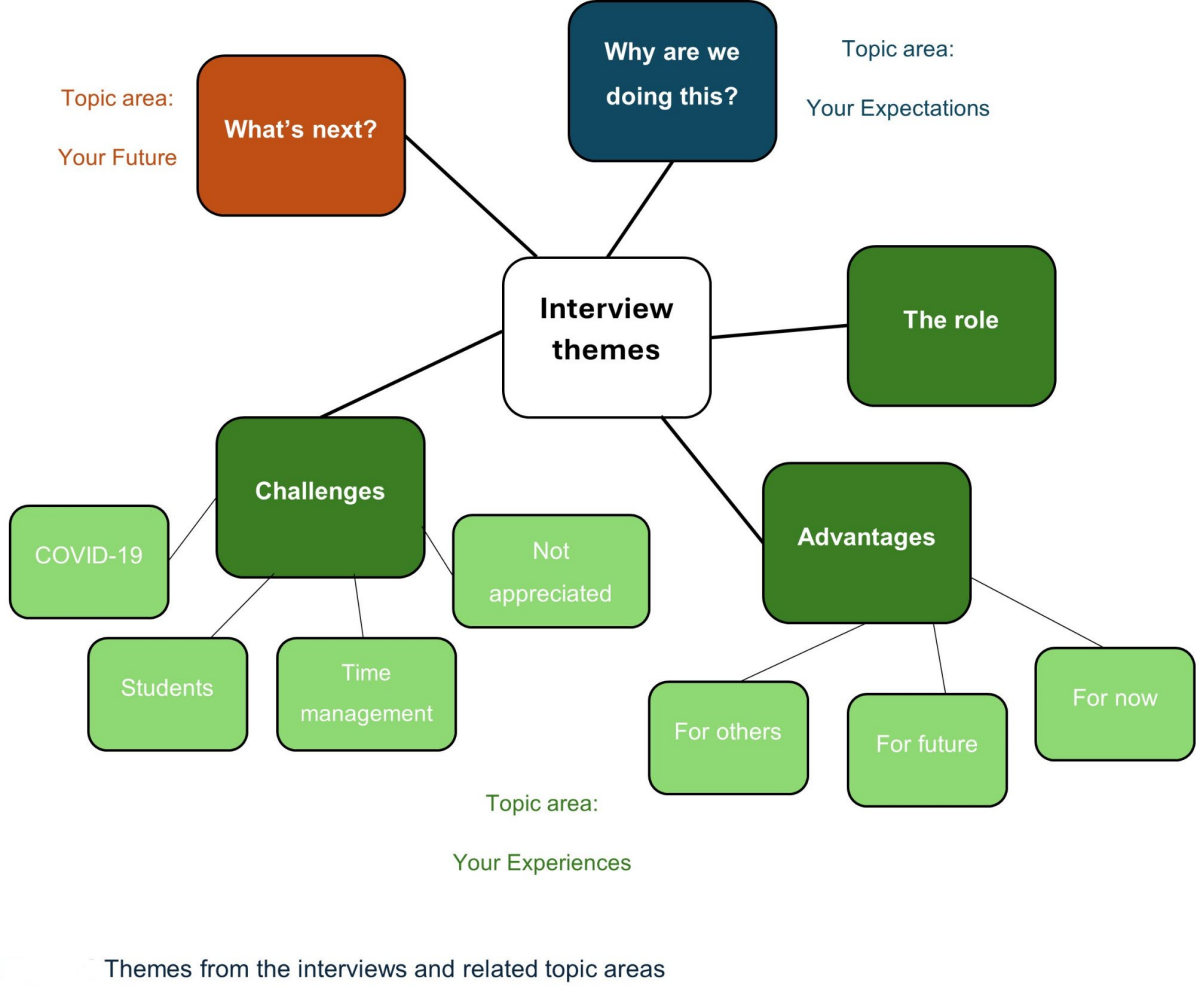
Themes from the interviews and related topic areas

Broadly, the first theme maps to the topic of Your Expectations as detailed on the topic guide, themes 2–4 map to the topic of Your Experiences, and the fifth theme maps to the topic of Your Future (Fig. 1).

Within this theme, participants discussed the reasons why they had chosen to spend the year as a CTF. Broadly, the reasons were related to teaching and taking a break from their usual job.

### Teaching

Having had contact with other CTFs through either being taught by them or helping provide teaching in their roles prior to becoming a CTF themselves was a reason given by several participants as one that had influenced their decision.

“I did lots of informal teaching on the wards, I enjoyed that and did like various bits of teaching through that time and always thought, I remember seeing Clinical Teaching Fellows come on to the ward and think like, oh, it would be great if that was your sole, your only responsibility for a bit, that would be really nice.” Participant 8.

For one participant, a key reason for wanting to spend a year as a CTF was their own experiences of receiving teaching and a desire to improve learning environments for students.

“I think one of the main things was having really good and really bad teaching at med school. I think wanting to be able to help, sort of, with the new generation of doctors, sort of instil a bit of better teaching, and you know, a bit of a nicer learning environment was definitely part of it.” Participant 6.

#### Taking a break

A key reason for applying to the post for several participants was the desire to have a break as they felt exhausted or burned out from what they had been doing. Having worked during the COVID-19 pandemic was a particular reason for one participant.

“When I applied for this CTF role, I had been redeployed from the breast team. I was COVID ICU in that first wave, I just, I was really knackered. I hadn’t been able to take any annual leave. I was quite fed up. And it’s been, it was a nice way to have a little break, actually. No nights, no evenings, no on calls.” Participant 4.

For another participant, they identified having gone straight through from foundation training to specialty training as a reason why they wanted to take a break.

“So part of the reason I’ve done this, so I’m post GP training. So I’ve done F1, F2 and three years of GP training, and wanted this role, because I wanted a break from clinical medicine. I was getting a bit burnt out having done, it sounds a bit silly, you’ve had just five years in a row but a lot of people do take breaks between F2 and specialty training. I didn’t and I have had a really tough time doing GP, it wasn’t for me.” Participant 8.

Two participants spoke more widely about why they thought people other than just themselves were wanting to take a break and do the CTF job. They identified the nature of postgraduate training and the demands of an on-call rota as key reasons.

“People getting tired of the on-call craziness and wanting a bit of a break. I think that’s probably the main reason a lot of people I know that have done the teaching fellow role. They’ve done it when they’ve had children or when they’ve just felt shattered from the on-call rota [laughs]. Which, yeah, which is, I don’t know, seems a bit of a shame really, that you get to the point where you’re shattered and you have to change your job just for a bit of a break.” Participant 2.

“I think it’s something like 80% of junior doctors don’t go straight into training or something like that, it’s very, very high. And I think that’s possibly a failure of the postgraduate medical training system is that we feel that we need to do that, that we need to take a year out to kind of have a bit of a break and to do some other things.” Participant 6.

For other participants, the desire to take a break was because they were unsure of which specialty to apply to and wanted more time to decide, or because they didn’t yet feel ready to embark on the next stage of training.

“I didn’t know what to do with postgraduate training. I didn’t know what to apply to. I was having a crisis because I thought I was going to do one thing and then decided, oh, maybe I should do another thing.” Participant 5.

### 1. The role

Within this theme participants discussed the day-to-day aspects of being a CTF such as duties associated with the role and specific aspects of their own posts, what starting the role had been like, and whether they felt their own experiences of the role had been typical of a CTF role.

#### Duties & specific post aspects

Whilst all the posts had responsibility for providing teaching, a large variety in the duties associated with individual posts was reported (e.g. responsibilities for teaching particular topics or particularly year groups.) A lack of standardisation was evident between posts with individual descriptions of the CTF roles highlighting specific differences with how their job was structured or created by the Trust.

“So I applied for 100%, like a full time CTF role and actually missed out on a job. And then one of the people who had been offered a job decided they only wanted to do three days a week, not the full five. So I was obviously next on the list, they came back to me and said, well would you like to do two days a week and we’ll make sure you still get paid for a full time job and you’ll do clinical work the other three days.” Participant 6.

As a result of this lack of post standardisation and as detailed in Table 1, not all participants were contracted to undertake clinical work on a mandatory basis. Participants broadly felt they were in roles that suited what they wanted to do, whether that was having clinical work contracted or not.

“And I’ve been fortunate that there’s no clinical commitment at [Trust name] it’s purely teaching. So that’s been really good.” Participant 8.

“Actually then having that change halfway through the week was really nice. I’d do three days of clinical, and be a bit bored and then go into teaching for two days and get a bit bored of it go back and forth. So I really enjoyed that.” Participant 6.

#### Starting the role

An aspect of the role that several CTFs discussed was the start of the job and how important the handover they had received had been. For some of the CTFs, someone who had been employed in the role had stayed on for another year and was therefore able to provide guidance for the new starters.

“We’re lucky that a couple of the fellows this year worked last year. And they knew the ropes, so when we started it wasn’t a completely blank slate, we had a good bit of structure to go with and we just fitted in.” Participant 3.

There was an acknowledgement that whilst having someone stay on could be beneficial, this wasn’t always the case and it was important to plan ahead to try to provide as much handover as possible for the incoming CTFs.

“And I’ve tried to set up for the next years so there’s a bit of a handover whilst we leave and they start. And I’ve left my materials in a shared drive and maybe they can at least reference and have a bit of an idea about what to do. I think that handover phase can be difficult.” Participant 4.

#### Typical experience

The participants were asked if they felt they had a typical CTF experience as part of the interviews. One participant was confident that they had had a typical experience and through having spoken to other CTFs didn’t think that there could be much variation in experiences between posts.

“I don’t see how it could be that different to be honest. I would imagine it’s pretty typical. From word of mouth, from those informal discussions you have and [name] and [name], the other CTFs, both have quite good friends that are in the other roles and my other half, he did the CTF role few years ago at a different Trust and it’s all fairly similar.” Participant 8.

For other participants, they felt that their jobs had been particularly busy, either through having a small team of CTFs at their Trust or through having a large number of students to teach. Whilst they felt that their experiences had broadly been typical of other CTF roles, they felt there had been differences resulting from this.

“One of the things I’d say that’s different talking to others through the PGCert, is their departments have a number of CTFs that is much larger than what we have here… So it’s, I think, what we do, the teaching and everything like that I think it’s very typical and the challenges we find and face with all of the teaching and the logistics, probably very similar. But I do think our workload is quite different to some of my colleagues. Definitely.” Participant 3.

One participant said they felt from talking to others that they had had a very different experience, but they weren’t sure what a typical CTF experience would be like.

“I mean, I don’t really know what a typical CTF role is. And, sort of, when I’ve been on my PGCert, like, hearing bits from the others, it does sound really different. They’re talking about different year groups and having students for like a longer period of time, and getting to know them for like weeks and things.” Participant 9.

### 2. Advantages

Within this theme participants described the advantageous aspects of the CTF role and these fell into three main categories; (a) For now, (b) For future, and (c) For others.

- For now.

Within this category participants described the advantageous aspects that had a direct impact upon them whilst they were in post. A key positive element identified with being a CTF was the lifestyle benefits that the job afforded in terms of job stability, allowing more time outside of work, and having a break.

#### Lifestyle benefits - job stability

Having the stability of a year-long contract working in the same team as opposed to the usual job rotations in foundation training allowed participants to have what they perceived as a ‘normal’ job experience and to feel a sense of control over their working lives.

“It’s the first time you’re in a team, and you’re at work in the same place very whole year. So you get to build those connections that normal people get to build in day to day life [laughs]. Whereas rather than being shipped off every four months, so that stability has been really nice.” Participant 8.

“Just do things that you don’t otherwise get a chance to do when you’re on what feels a bit like a conveyor belt a lot of the time with training and being you know, sent all across the region all the time. It was nice to kind of take control for a year.” Participant 2.

#### Lifestyle benefits - time outside of work

Participants identified working regular day time hours and not working weekends as a particular benefit of the CTF role.

“I think the lifestyle things are really nice. I mean, that’s obviously a big pull, the fact that it is, you know, nine to five, Monday to Friday, and okay, sometimes I need to prep a lecture, but mostly I can leave my work and that’s that. There are no on call day weekends, it gives me time to do my extra bits and bobs.” Participant 4.

Some participants detailed specific life events/family commitments that had been made easier by working regular hours and having more time outside of work.

“But I think [husband] really enjoyed me doing the CTF year as well, just because he saw me loads more. We weren’t constantly thinking about what we’re going to do with the kids and, you know, having to plan every single weekend lots in advance and stuff. So for family life, it’s great.” Participant 2.

#### Lifestyle benefits - having a break

Several participants identified having a break from particular elements of working as a doctor, such as on call rotas and being on a training pathway as a particularly positive aspect of the year.

“And I think, for me, it’s been quite a nice year, just finished foundation training and kind of gone into a nice year of being able to still do something medical and keep in that field but be out of training for that year as well.” Participant 7.

#### Job enjoyment

Another advantageous element identified was the sense of job enjoyment that was derived. Participants described enjoying the different elements of the job (e.g. providing teaching, having protected time for teaching, seeing students develop over time, providing pastoral support), and enjoying going to work.

All of the participants said they had enjoyed their job over the year and detailed how enjoyable the teaching aspect was. Many of the participants detailed having done some teaching in previous roles, but having protected time for teaching in their CTF role was a factor that was identified as being particularly enjoyable.

“For myself, I’ve always loved teaching so it kind of gave me an opportunity to focus on that a little bit more. So within your foundation years you do manage to get some teaching in but it’s kind of it is in between your working days or when you can, whereas this gave a little bit more opportunity to experience it.” Participant 7.

For some, the year had exceeded any expectations they had had prior to starting, and for one participant this was particularly to do with being able to make their own decisions about how they taught and the enjoyment they had derived from that.

“I think it’s exceeded my expectations. I don’t know what I thought going into it. I think I knew I’d be teaching, I knew I’d be doing different forms of teaching, but it’s definitely sort of exceeded my expectations in the sense of getting such a breadth of it, being able to and having that artistic licence to be able to kind of do what you want, within reason.” Participant 8.

Other participants also reported enjoying being able to create their own sessions and have input into designing teaching. Participants described how they had been able to reflect on their own teaching style and make changes, or had identified topic areas that others were struggling with and been able to design sessions based on that need.

“So I think being able to plan and prep my own teaching sessions has been quite a positive thing, because I’ve been able to look at different ways of how I can do perhaps the same session.” Participant 7.

Participants also described how rewarding they had found teaching, and how they had enjoyed watching students develop over the time they taught them.

“With the students, what’s really nice is actually because you work with them for a period of time, that feedback, it just constantly builds and builds and it’s really good to see that student develop who at the start term was very nervous, but actually now they’re really confident.” Participant 3.

Another element of the job that had been enjoyable for some participants was providing pastoral support for students. One participant said this was a more unexpected aspect of the role but one they had liked.

“A surprising but also really nice aspect of the job is having that sort of mentorship pastoral role. And because you know that all of those more formal processes are in place, so because you know that there’s a senior academic tutor and you know, that there’s the student services desk at uni and things, you don’t feel out of your depth in managing it. So that was a really nice part.” Participant 8.

Others said they enjoyed receiving feedback on their work, and one contrasted their experiences of this with working in clinical medicine.

“You get lots of nice comments and [feedback] you can start to apply, which is something that doesn’t really exist in clinical medicine so much. Often it’s you’re only told the things that haven’t gone quite so well and the things you need to chop and change slightly.” Participant 3.

In addition to the teaching aspects, having a nice work environment was highlighted as a particularly enjoyable aspect of the year for one participant.

“You know, I’ve been really lucky, I’ve got some really nice colleagues, we have a bit of a laugh, and the admin stuff as well, we get on well, so I actually look forward to going to work in the morning, it’s quite nice.” Participant 4.

b) For future.

Within this category participants described the advantageous aspects that would have an impact upon them in their future careers in terms of developing skills and being able to enhance their CVs. This included personal development in terms of gaining teaching qualifications/skills, as well as being able to explore areas of interest outside of medical education.

#### Teaching qualifications/skills

An aspect of the CTF role that all participants identified as beneficial was the opportunity to undertake a funded postgraduate educational qualification (in most cases this was the PGCert offered by UoB, but CTFs who already had this qualification had the opportunity to undertake the PG Diploma instead). Some participants said the PGCert had been useful to do whilst they were in post as it helped them to develop as a teacher.

“I could really see how the PGCert helped me throughout the year as well. It was nice to be able to apply in the sessions we had on things for group teaching and how to work in with difficult groups and stuff, it was all was really useful, really nice to be actually doing the PGCert alongside it. It was a relevant syllabus.” Participant 2.

Several participants said being able to undertake the PGCert qualification was a key reason why they had decided to do the CTF role.

“But I think I could speak for all of the teaching fellows I’ve worked with here when I say if the job didn’t offer the postgrads certificate in medical education, I probably wouldn’t do it.” Participant 3.

Participants highlighted the importance of being able to demonstrate having teaching experience when applying for future specialties, and indicated that the CTF role is an excellent way to enhance applications. This was identified as an advantageous aspect that may motivate people to apply for a CTF post.

“There is more of an emphasis on getting those audits and QIs and teaching points [for specialty applications]. And it’s not just about being a good doctor anymore. You have to have publications and presentations… I’ve got so many points I was in the top 1% of the UK. So it’s, yeah, I wasn’t expecting that. But it’s having those points, actually doing things like the teaching and the CTF year, it actually does make a difference.” Participant 4.

#### Explore areas of interest

The CTF year also gave post holders opportunities to gain experience in their specialty of choice. This was done for several reasons including CV enhancing to assist with future specialty applications and helping to decide if a particular specialty was what someone wanted to pursue long term.

“What one of my colleagues has done is last year, they were full time as a Clinical Teaching Fellow, this year they’re part time. And so the other half of their time is clinical in an area they’re interested in. And I know that they’ve now applied for that specialty, and when you look at their ranking in the country for where they rank to get into that specialty, they came very high. And there’s no doubt that actually the last 12, 18 months is what did that for them because they were just able to pick up so many bits to add on the CV and experience, exposure.” Participant 3.

For other participants this was seen as a chance to take some time to do things that people wanted to do out of choice, rather than having to do what was dictated by a formal training programme.

“Training can be a little bit like I’ve said already, like you’re on a conveyor belt just ticking the boxes? Whereas it was nice to do something to further me rather than ticking boxes for somebody else if you see what I mean?” Participant 2.

c) For Others.

Within this category participants described the aspects of the role that had a positive and advantageous impact upon others. These related to the students they taught and the Trusts which employed them.

#### Students

The participants felt that the benefits of CTF teaching for the students were being able to teach more relevant topics and being able to teach what the students wanted to learn. The CTFs reported that the students were interested in learning what they needed to know to (a) pass their exams, and (b) be a safe and competent foundation doctor, and the participants felt that as they were closer in age to the students than the consultants and because they had recently been in the same position as students, they were more able to offer what students wanted.

“I guess having a qualified doctor whose sole job is to teach a student, I think there’s a huge value in it being, it’s not quite near peer, but we get a lot of feedback and comments that say you’re closer in age, you know our curriculum better, you’re a Birmingham grad this, that and the other, and we find that more valuable than consultants teaching who are very experienced in their field” Participant 8.

Related to this was the CTFs reporting students finding them approachable. They thought was beneficial as this helped with students learning and being able to ask for help with things they were finding hard.

“I think this idea of CTFs being closer in age has meant they are more approachable. And if you’re perhaps worried about something, or you’re not quite understanding something, you’re more likely to approach one of the CTFs and say, actually, I don’t quite understand this, as opposed to perhaps approaching a consultant.” Participant 7.

A further positive aspect of CTF teaching for students was the reliability they felt they could offer through the protected teaching time built into their job.

“This is our job, we are dedicated to that teaching time. So if we say we’re going to be teaching from one to two, we will teach them from one to two, whereas consultants, registrars etc., will have clinical commitments that may pull them away from the teaching time, despite it meant to be part of their PAs, protected etc. So I think [medical students] find us sort of more reliable about doing it.” Participant 5.

#### Trusts

The participants identified the positive aspects of CTFs for the Trusts as having a group of employees who were able to take pressure off clinical staff to teach and fill in for consultants who were facing time pressures in delivering teaching.

“I think because some of the consultants aren’t doing as much consultant teaching as maybe, or they don’t have the time to do as much consultant teaching as they did because, I don’t know, whether it’s work pressures or preferences or what, what drivers are there, but because they perhaps can’t afford to do as much, we’re sort of stepping in and taking the slack.” Participant 4.

### 3. Challenges

There were four categories of challenges described by the participants. These were (a) COVID-19, (b) Students, (c) Time Management and (d) Not Feeling Appreciated/Listened To.

#### A) COVID-19

All of the participants detailed how the COVID-19 pandemic had impacted upon their job in some way. Some participants were redeployed to clinical work full time during waves, whilst others remained as CTFs but were required to provide cover for colleagues who were on sick leave where needed. The biggest impact reported upon teaching was the duplication of sessions required to ensure that smaller group size numbers and social distancing requirements were met, and the impact this duplication of sessions had on preparation time.

“COVID has put a big spanner in the works with a lot of things, so bedside teaching groups being smaller has meant we’ve had to duplicate our time So the prep time is significantly more because you’re kind of preparing two different patients on two different wards.” Participant 8.

Additional challenges related to COVID included not being able to have as much contact with other CTFs working in different Trusts as participants would have liked. This had an impact upon being able to share teaching ideas, socialising, and offering peer support.

“The only way of contact with the CTFs is either if you happen to know them by chance, or through the through the PGCert. And I think previous years have been able to have that chit chat over ‘what do you do with the 3rd year? What do you do?’ and know that, whereas we’re only getting that from the students, because we don’t, because everything’s online, it’s different, more difficult to have those informal conversations with the other CTFs.” Participant 8.

#### B) students

Some participants at the larger Trusts described how the number of students placed there presented challenges when providing teaching. Large numbers of students meant that the CTFs were having to deliver high volumes of teaching which they found overwhelming. This was an issue exacerbated by COVID and the associated restrictions.

“Related to student numbers, we’d had to change the way that we taught quite sort of considerably because of COVID, and the number of students and things like that. So for us, it felt like we were doing an enormous amount of teaching, because we were basically having to do everything sort of six, seven times to get all the students through. So obviously, for each individual student, they’re only been having one of those interactions with us. So for them, it feels like they’re not having contact with us as much. Whereas we are feeling quite overwhelmed by actually how much teaching we’d have to be doing.” Participant 6.

For some participants, having to deal with student behaviour was an unexpected challenge. One participant felt this was a particular problem as large student numbers made it difficult to build rapport with individuals, whereas others felt closeness in age made this difficult.

“I think the main challenges were sometimes having difficulties with rapport with the students, which I wasn’t really expecting, and sometimes having to… discipline isn’t the right word, because obviously, that’s not really the role that we have with them, but be a bit firm with them and actually explain to them when they’ve done things wrong, I found that really challenging. And it just hadn’t really occurred that that was something I was going to have to do.” Participant 6.

#### C) time management

Time management was an area identified as a challenge by several participants. This was a particular issue at the start of the year and resulted in participants having to work outside of their contracted hours.

“I think for all of us at the start, we were finding our feet and figuring out what, what we, how to manage our time. I know when we all started, we found balancing kind of working and prepping and the admin side of things. So there were times where you would go home and be working for a few hours each evening, as well as having taught all day.” Participant 7.

One participant detailed how managing their time was a challenge as the job was so busy, and this resulted in certain aspects of personal development having to be sacrificed to fulfil the requirements of the job.

“Time management has been a big challenge because there is so much to cover. And I think just trying to make sure that you’re staying ahead, because you do want to still have your… obviously teaching comes first but you do still want to have that clinical aspect. Trying to negotiate no we can’t take on another three, four days of sim teaching, because actually, our timetable is already full and I’m already struggling to get my clinical days in as it is. Erm…that’s been quite difficult.” Participant 4.

#### D) not feeling appreciated/listen to

For one CTF, not feeling listened to or appreciated was an aspect they found challenging during their year in post. They described how they had felt like the senior members of staff at their Trust had not listened to their suggestions or opinions on teaching matters.

“I think one of the other frustrations I had this year, it kind of ties in to not being listened to by the senior team a little bit. And I don’t know if this is just a foible of the hospital I work in or if this happens to other CTFs as well, but I think, definitely, we sometimes felt a little bit infantilized by the senior team as though we were sometimes naughty school children that needed telling what to do certain things, and they didn’t, again, didn’t listen to our opinion on things. And when we sort of suggested things didn’t really listen.” Participant 6.

Particularly for this participant, the frustrations around not being listened to centred around the level of expertise in medical education that the CTF team had vs. the senior members of staff at the Trust.

“We’re grownups who have a very, very good understanding of teaching and what aspects are involved, especially something, so three of our CTFs are doing the PGDip, so they’d already done the PGCerts, they have a very good collaborative understanding of medical education theory and everything like that, and just not being listened to was very, very frustrating by these sort of senior doctors who quite often, a lot of them were actually new to the post this year so didn’t have any more experience, lots of them had even less experience than we did, within sort of Medical Education Department.” Participant 6.

They also felt that the medical students didn’t appreciate how much time and work the CTFs had to put in to preparing their teaching sessions as they made requests for extra resources without realising the additional work that would be required to provide them.

“And sometimes the amount of effort they expect us to put in, I don’t think they realise actually how much stuff goes on behind the scenes to sort of actually get that done. And so they’ll be like, Oh, well, why can’t you just make a set of notes that we could have for this session, not realising that that’s probably actually several hours worth of work to sort of compile a set of notes to go with a session for them? Why can’t you just do this extra work?” Participant 6.

### 4. What’s next

Within this theme, the participants described their plans for the future and what their involvement with medical education would look like. This included plans for the immediate next steps of their career and what they thought their long-term career plans would be.

### Immediate future

The majority of participants indicated a desire and plan to keep on teaching in their immediate next jobs. This was with varying degrees of formality from hoping to be able to offer teaching in spare time to having a designated voluntary role at the Trust. For a lot of the participants planning and co-ordinating their time, staying at the same Trust for their next job and/or building and maintaining good working relationships were seen as a key factors in facilitating future teaching involvement.

“So I’m conveniently actually staying in the same Trust I’ve been in for F1, F2, and CTF. So I probably will carry on teaching. They have what they call junior associate teachers at [Trust] where we’re junior doctors, but we have like a group of students, and they’re the ones that you go and do the bedside teaching with them. So I’ll probably volunteer to do that again, and volunteer and come back and do a few sessions.” Participant 6.

One participant indicated that if it had been possible to stay on as a CTF and also progress their career they would have done that.

“I would have stayed on if I could have progressed and continued my core medical training and my reg training, if I had a protected day or two a week to do it, I would have. I would have lapped that role up. I’m just sorry it doesn’t exist [laughs].” Participant 4.

They went on to describe what their ideal immediate future job would have look like, where they would be able to progress their clinical career and also be able to continue having formal involvement with medical education without having to take a break to do so as they had done with their CTF year.

“It’d be really nice if in the future, there was a role in the medical training route where you kind of, I know they’ve got academic clinical fellowships, where, you know, obviously, some of that’s research and some of that is, if you do Med Ed you might have protected teaching time. But it would be nice if there was more protection in that future role, in your clinical training to do teaching to do, you know, maybe it was a dual role or Med Ed and Medicine or something. I don’t know.” Participant 4.

### Long term career goals

All of the participants were keen to have a senior job in the future that involved education. There were varying degrees of planning set out, for some, it was just an idea that they would like to continue involvement in medical education but they weren’t entirely sure what this would look like or entail.

“My desire is that when I qualify, become a consultant, I think I would like to make teaching as part of my career.” Participant 1.

Other participants, however, had made firmer plans and were actively pursuing a speciality (general practice) they had identified as one that would enable a flexible career involving teaching.

“And my overall goal is to have my role as a GP, but also have a role in education, whether that be undergrad or post grad education so to kind of split my time between the two would be my goal for the future, I guess. It is definitely more flexible from what I gather, in general practice to be able to, you know, take, for example, three, four days in clinic and then the other one or two days in education.” Participant 7.

## Discussion

This study set out to interview CTFs at the end of their time in post to ask about their experiences. It found reasons for choosing to spend a year as a CTF included wanting a break from clinical work and having previously enjoyed delivering teaching in prior roles. It also found the role had delivered advantageous aspects related to the reasons for choosing the job as well as offering opportunities for self-development, and there was a desire from participants to have continued involvement with medical education in their future careers. The data highlighted five themes which were related to expectations of the post, experiences in post, and future careers. The main findings of the interviews are discussed in detail below.

A key reason given for why participants had wanted to take up a CTF post was wanting a break from the exhausting nature of clinical work and/or the postgraduate training pathway. It was evident from these interviews that the CTF posts had met participants’ expectations particularly in respect to wellbeing with participants detailing advantages of the role such as having a better work-life balance and enjoying more time outside of work. The importance of work-life balance and life-style permitted by specialty choice has been documented [24, 25], and it appears that these factors are similarly important when choosing to take a year out of training. With the majority of junior doctors now taking a break after the first two foundation training years and citing reasons for doing so such as wanting time out from the pressures of a training pathway and feeling burnout [2], this is clearly an important issue in postgraduate medical training in the UK. Two participants in this study particularly highlighted this through saying either that they felt it was a shame that a job change was required to have a break or that needing to take a year out to have a break was a possible failure of the postgraduate training pathway. This is an area clearly warranting future research, particularly with wellbeing and retention of workforce now being seen as a critical issue facing the NHS [10].

The other key reason given by participants for taking up a CTF post was having had prior contact with other CTFs and experience of delivering teaching. Having either been taught by CTFs when medical students themselves or working alongside CTFs to provide teaching had given participants an idea of what the job would be like and confirmed this was something they would like to spend a year doing. This reflects what is already known about the influence positive role models can have on career/specialty choice in medicine [26, 27]. It could be that the increase in number of CTF posts is somewhat driving itself as more medical students than ever before are being exposed to CTF teaching and given access to potential career role models that would not have existed previously.

As participants in this study had selected a role with a teaching component based on an existing interest in teaching, it is unsurprising they enjoyed this element of the job. However, the direct comparisons drawn between their enjoyable experiences in the CTF role and clinical work are of interest, particularly with regards to feedback received and the continuity/stability of their role. Two participants detailed how it was nice to receive feedback they could act on and apply rather than only being told things that hadn’t gone well, and how working with the same group of students over time meant they could apply the feedback to their teaching and they found it rewarding to watch students develop over time. The continuity and stability of the role was commented on by another participant who said how nice it was to work in the same team for a year and be able to build more connections with team members than they usually were able to in their clinical roles. The starkest contrast between the CTF role and clinical work however was the participant who said they had actually looked forward to going to work as a CTF, implying this was not always the case in their clinical roles. These findings are relevant to the previously mentioned issue of wellbeing and retention of workforce, and future work exploring this area may be beneficial.

Supporting findings in previous research [7], there was variation in the duties ascribed to the individual posts held by the participants in this study, and particularly with the time allocated to clinical work. This lack of standardisation of CTF posts, also apparent through Trusts being able to create bespoke posts for certain participants, seems to be beneficial with the majority of participants reporting enjoying their posts and feeling like the time allocated to teaching or clinical work in their job suited what they wanted. For example, one participant reporting feeling ‘fortunate’ to not have any clinical commitment whereas another enjoyed having the mix of clinical and teaching work in their week. The participant who missed having contracted clinical work in their job said that if applying a similar job in the future, they would choose one that had a clinical element. Being able to choose a post that matches what an individual wants to get out of their CTF year seemed to be an important factor for those applying to the post. This was further reflected where participants described what they had found beneficial in their year such as being able to gain experience in a specialty or carrying out activities that would enhance future job applications. Gaining points for job applications is of particular relevance as the competition for specialty training places has been increasing [28, 29] and as noted by the participants, it is the extra activities that are important for scoring well. This suggests that lack of standardisation is a beneficial and advantageous aspect of the CTF role, and the ability to have choice over work duties/role extras is attractive and important to those considering becoming a CTF.

It is interesting to note that despite the variation in duties between posts reported by participants, broadly the participants felt their experiences had been typical of other CTFs. Participants perceived differences in the number of students taught or how busy they felt their particular post was, but with the exception of one participant, no major differences were highlighted. This suggests that the lack of standardisation is perhaps an expected and attractive element of the role to prospective CTFs who are looking to choose a role that meets their expectations and desires for the year.

The lack of standardisation and formalisation of the CTF role however could be a contributory factor to the way the role is viewed by other members of staff and students. For the participant who felt like the senior staff did not fully listen to the opinions of CTFs, formalisation of the role and recognition of the expertise in medical education that CTFs have may have changed their experience of this. Other participants reported that students did not seem to show CTFs the same level of respect as they would towards consultants through issues with punctuality or professionalism. They identified that the smaller age gap between the students and the CTFs was beneficial in building rapport and offering teaching similar to near-peer teaching [30], but this could perhaps be where the problems arose, as near-peer teaching is also known to have disadvantages such as teachers having a lack of authority over students [30]. It could be that formalisation and recognition of the role becomes necessary to remove challenges such as these as CTFs are now increasingly taking on the majority of hospital-based undergraduate medical education.

Whilst some aspects of teaching students were raised as challenges by the study participants, the pastoral care element of the role and continuity of teaching the same group of students over time was detailed as something they found enjoyable and rewarding. Participants in this study feeling able to provide support for pastoral issues contrasts with findings from a previous study where CTFs felt that providing pastoral care was a significant challenge [7]. It would be pertinent for those employing CTFs to be aware that students are likely to bring pastoral issues to CTFs as a result of the amount of time they spend with them and the good relationships that are fostered through this teaching and therefore ensuring that CTFs have appropriate training and feel supported in detailing with such issues should be considered.

All of the participants in this study indicated they would like to have a senior job in the future that involved education, and the majority indicated that they would continue with teaching in their next jobs, but this was going to be through volunteering rather than having a formal role. One participant said they would like to continue in a similar CTF role and also be able to continue their core medical training at the same time, but they acknowledged that such a role was not available. There are some initiatives starting such as the NIHR Incubator for Clinical Education Research [31] that may be relevant to CTFs with this ambition, however these are more aimed at those who want to conduct educational research rather than teach.

It would be of interest to know how having held a CTF post impacts upon the future career of doctors and choices made regarding career path taken post-CTF role. As stated above, the participants gave indications of how they would continue teaching and it would be worthwhile to explore how the experience of a CTF post shapes teaching involvement once doctors have returned to the specialty training pathway. Future longitudinal research with former CTFs would be of benefit here.

It should be of interest that there is now an increasing group of junior doctors who have carried out a CTF role and gained a postgraduate qualification in medical education, but have no obvious route to continue in medical education [32]. With planned expansion of medical student numbers [9, 10], an increase in hospital-based teaching staff will also be required to provide adequate levels of teaching, and the participants in this study identified already that large student numbers provided a challenge for them. Whilst there are existing calls for the medical teaching workforce to be prioritised to ensure future generations of doctors can be taught and trained [33], this is focusing on academic clinicians who work in both universities and hospitals rather than hospital-based educators. Clinicians who are interested in academia have an established training pathway available to them encouraging research related to their chosen specialty and an expectation they will additionally deliver teaching [34]. It may be beneficial for future workforce planning to explore the options for the creation of a similar pathway for those who have shown an interest in hospital-based medical education, but not research, and wish to build a career in this alongside their chosen specialty.

### Strengths and limitations

This study adds to the very limited published literature exploring the CTF role and is the first to do so using interview methodology as part of a wider longitudinal study. Conducting interviews with CTFs at the end of their time in post (or in the case of 2 participants, a year after their time in post) enabled participants to reflect on their year and discuss how their expectations for the role had been met or not. The interviews have provided insights into the experiences of CTFs, explored why doctors apply for this job and what their future career plans are after completing a CTF post.

This study does have some limitations however. Firstly, not all of the invited participants from the wider cohort study took part in the interviews. This means the results may not be representative of those who took part in the other aspects of the study, and those who declined to take part in an interview may have had differing experiences to those who participated. The characteristics of the participants in this study do however show variation in terms of full/part time working, length of time after qualification, and Trust worked at.

Secondly, this study had a small number of participants which limits the findings. Ideally there would have been a large number of potential participants that could have been invited and agreed to take part in the study, and interviews would have been carried out until reaching data saturation, where no new data was being generated [35]. With a small predefined population opportunistic sampling was undertaken as is appropriate in such a situation [36], but attempts were made to maximise recruitment e.g. by also inviting participants from the first year of the study to be interviewed in addition to those from the second year of the study. It is suggested that 12–20 participants are required to reach data saturation [35, 37], and therefore this study does not meet this threshold. However, it is acknowledged that not reaching saturation does not invalidate the findings of a study, but rather suggests that further exploration is merited [38] which is an appropriate conclusion for this study.

Thirdly, there is a geographical limitation to this study with participants working in a selection of Trusts across the West Midlands region. However, medical education in the UK is nationally regulated by the General Medical Council and delivered within the NHS so it is therefore likely the results of this study would be applicable to other regions of the UK and possibly to other countries with similar models of undergraduate medical education. This work will be useful therefore to those employing CTFs in the West Midlands region and beyond, and as a springboard for those considering further research in this population in other areas of the country.

## Conclusion

This study has provided the first insight into experiences of CTFs using interview methodology and has enabled exploration of reasons why doctors choose to spend a year in a CTF role and the advantages and challenges of the role. It is evident the CTF role is an attractive job for doctors wanting a break from postgraduate training with its advantageous elements of offering a better work-life balance, no anti-social working patterns, and opportunities for self-development and exploration of areas of personal interest. The lack of standardisation, allowing for such self-development opportunities, was seen as a beneficial and expected element of the role, but it could be contributing to some of the challenges experienced such as lack of respect from students or lack of recognition of expertise from senior staff members.

With the continuing increase in numbers of medical students and therefore increasing requirement for staff to teach them, considerations for how to secure sufficient teaching staff should be of great importance. It is likely the CTF role will continue to be important in the provision of undergraduate medical education and therefore those employing CTFs should be aware of the reasons why doctors apply for CTF posts outside of an interest in teaching. This will help to ensure the post remains a desirable option and therefore help to ensure demands for teaching staff are met. Looking to the future and for longer term provision of medical teaching workforce, it would be beneficial for further work to explore how to utilise the increasing number of doctors with postgraduate medical education qualifications and accommodate those with an interest in building part of their career in medical education after completing a CTF post.

## Electronic supplementary material

Below is the link to the electronic supplementary material.

Supplementary Material 1

## Data Availability

The datasets generated and/or analysed during the current study are not publicly available due to participant confidentiality but are available from the corresponding author on reasonable request.
