# Supplementary material for: Experiences as a clinical teaching fellow: interviews with clinical teaching fellows in the West Midlands
Source: BMC Med Educ. 2024 Sep 16;24:1015. doi: 10.1186/s12909-024-05958-2 (PMC11406710; doi:10.1186/s12909-024-05958-2)
Supplement: Supplementary file 1 — Supplementary Material 1 [file 12909_2024_5958_MOESM1_ESM.docx]

**Appendix 1**

**CTF Interview Topic Guide**

Your experiences

- Can you tell me about your year in post?
- What were the positive aspects of the role?
- What were the challenges of the role?
- Do you think your experience of the role was typical of other CTFs?
- Do you think the role will change going forward?

Your expectations

- Why did you want to do the role?
- How did the role meet your expectations for the year?

Your future

- How do you think this role will impact upon your future career?
- Do you intend to have continued involvement in medical education, and how so?
